# Contact and Encirclement of Glioma Cells *In Vitro* Is an Intrinsic Behavior of a Clonal Human Neural Stem Cell Line

**DOI:** 10.1371/journal.pone.0051859

**Published:** 2012-12-11

**Authors:** Nousha Khosh, Christine E. Brown, Karen S. Aboody, Michael E. Barish

**Affiliations:** 1 Department of Neurosciences, Beckman Research Institute of the City of Hope, Duarte, California, United States of America; 2 Department of Cancer Immunotherapeutics and Tumor Immunology, Beckman Research Institute of the City of Hope, Duarte, California, United States of America; 3 Division of Neurosurgery, Beckman Research Institute of the City of Hope, Duarte, California, United States of America; The University of Chicago, United States of America

## Abstract

Pathotropic neural stem and/or progenitor cells (NSCs) can potentially deliver therapeutic agents to otherwise inaccessible cancers. In glioma, NSCs are found in close contact with tumor cells, raising the possibility that specificity of NSC contact with glioma targets originates in the tumor cells themselves. Alternatively, target preferences may originate, at least in part, in the tumor microenvironment. To better understand mechanisms underlying NSC interactions with glioma cells, we examined NSC-target cell contacts in a highly simplified 3-dimensional peptide hydrogel (Puramatrix) in which cell behaviors can be studied in the relative absence of external cues. HB1.F3 is an immortalized clonal human NSC line extensively characterized in preclinical investigations. To study contact formation between HB1.F3 NSCs and glioma cells, we first examined co-cultures of eGFP-expressing HB1.F3 (HB1.F3.eGFP) NSCs and dsRed-expressing U251 glioma (U251.dsRed) cells. Using confocal microscopy, HB1.F3.eGFP cells were observed contacting or encircling U251.dsRed glioma cells, but never the reverse. Next, examining specificity of these contacts, no significant quantitative differences in either percentages of HB1.F3 NSCs contacting targets, or in the extent of target cell encirclement, were observed when HB1.F3.eGFP cells were presented with various potential target cells (human glioma and breast cancer cell lines, patient-derived brain tumor lines, non-tumor fibroblasts, primary mouse and human astroglial cells, and primary adult and newborn human dermal fibroblasts) except that interactions between HB1.F3 cells did not progress beyond establishing contacts. Finally cytoskeletal mechanisms employed by HB1.F3.eGFP cells varied with the substrate. When migrating in Puramatrix, HB1.F3 NSCs exhibited intermittent process extension followed by soma translocation, while during encirclement their movements were more amoeboid. We conclude that formation of contacts and subsequent encirclement of target cells by HB1.F3 NSCs is an intrinsic property of these NSCs, and that preferential contact formation with tumor cells *in vivo* must therefore be highly dependent on microenvironmental cues.

## Introduction

Despite improvements in conventional therapies, the prognosis for patients with glioblastoma remains dismal in part due to recurrence seeded by disseminating tumor cells. Effectively targeting invasive cells and microfoci would be a significant therapeutic advance, but remains technically problematic. The intrinsic tumor tropism of neural stem cells (NSCs) [1], [2] is a physiological mechanism potentially exploited for delivery of therapeutic agents to otherwise inaccessible tumor foci [3]–[6]. Towards this end, a number of clinical trials related to brain cancers have been initiated or are in preclinical development [7].

Tumor tropism is a property of both endogenous [2], [8], [9] and exogenously expanded (including immortalized) NSCs [1], [10], [11]. NSCs implanted intraccranially (i.c.) ipsilateral or contralateral to orthtopically engrafted gliomas follow perivascular spaces and white matter tracts, while NSCs introduced intravascularly (i.v.) extravasate at tumor sites [12]. In either instance, NSCs ultimately localize to and associate with tumor masses. These complex processes of necessity involve multiple environmental cues including soluble factors and extracellular matrices. While the signals guiding tumor-directed migration of NSCs are not fully identified [13]–[18], long distance NSC homing *in vivo* appears to be selective for tumor cell targets. Comparisons of potential targets have shown migration in response to various types of brain tumors and not, for example, fibroblasts [1], [8]. At the same time, NSCs also migrate towards sites of injury, ischemia and inflammation [8], [19]–[21], suggesting that migration *in vivo* may be dependent on cytokines and signals originating from both tumor cells and the host tissue reactions they elicit in surrounding brain [22], [23].

One unresolved question of therapeutic significance involves the formation of close contacts between NSCs and tumor cells within the brain parenchyma [1], [10], [11], [24], [25]. These observations, reported in multiple studies, raise the possibility that preferential formation of NSC contacts with glioma cells may be a reaction to intrinsic properties of tumor targets. An alternative possibility to consider is that subsequent to long-range NSCs migration, preferential tumor cell contact selectivity may be a response, at least in part, to signals present in the tumor-altered microenvironment. Evaluating these alternatives and understanding the fundamentals of NSC-tumor interactions at the level of individual cells may contribute to optimizing NSC-based therapies, including tracking of disseminating tumor cells.

To better understand the mechanisms underlying NSC interactions with tumor cells, we examined NSCs and target cells in the absence of surrounding brain and its microenvironment. In this highly simplified 3-dimensional peptide hydrogel, environmental signals normally present in the brain will be at a minimum and cell-cell interactions can be studied in relative isolation. We focused on the clonal human NSC line HB1.F3 that has been extensively studied in preclinical investigations and is presently in clinical trial for treatment of patients with recurrent glioma (www.clinicaltrial.gov, NCT01172964). HB1.F3 cells are immortalized, clonal, non-tumorigenic and minimally immunogenic cells that express the stem cell markers nestin and Musashi-1, and exhibit multi-lineage differentiation *in vitro* and *in vivo* [26], all characteristics of neural stem cells.

## Results

### Morphology and movement of interacting human HB1.F3 NSC and U251 glioma cells

To examine HB1.F3 NSC movements and interactions with U251 glioma cells, previously studied in *in vivo* mouse models [12], in the absence of the brain microenvironment, we employed a synthetic peptide hydrogel (Puramatrix) [27] into which HB1.F3 NSCs and target cells were encapsulated and cultured (see Materials and Methods).

**Figure 1:**
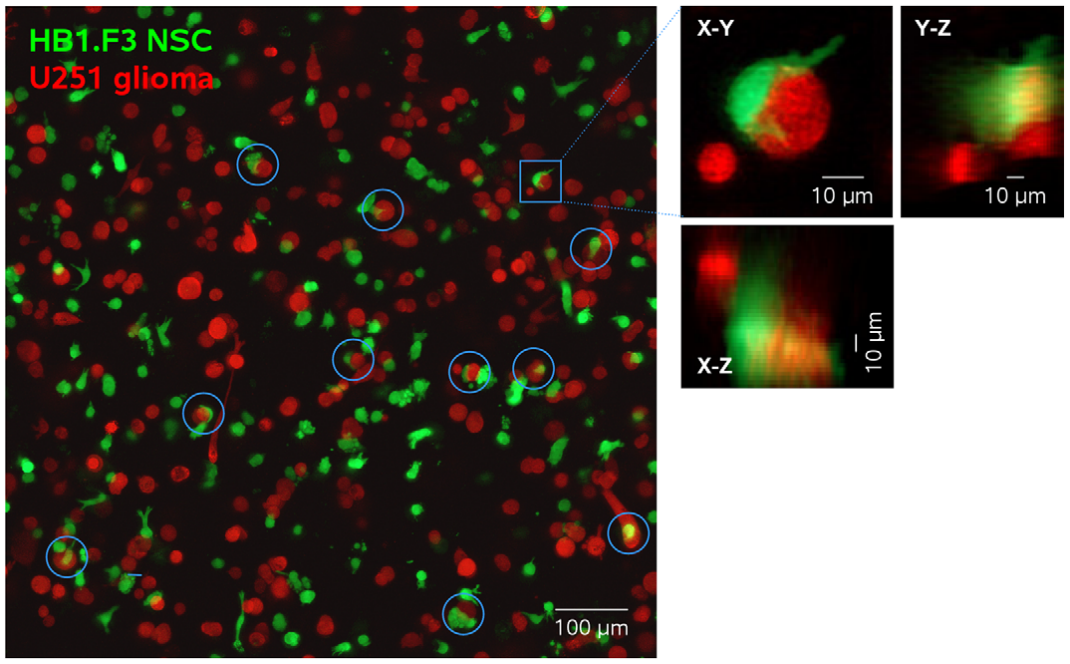
Spatial relationships between HB1.F3.eGFP NSCs (green) and U251.dsRed glioma cells (red). Cells were co-cultured for 18 h in 3-dimensional Puramatrix hydrogel. Shown is the Z-axis projection of 34 optical sections of 900×900 µm spanning 102 µm. Expanded X-Z projections of individual contacting cell pairs confirm true contacts rather than superposition. Of a total of 157 HB1.F3.eGFP cells in this image, 38 (36.9%) were contacting U251.dsRed cells. Of the remaining solitary cells, 94 (59.9%) were roughly spherical or ellipsoid with minimal or no process extension, and 25 (15.9%) showed extended filopodia-tipped processes.

We first examined the interactions of eGFP-expressing HB1.F3 (HB1.F3.eGFP) NSCs and dsRed-expressing U251 glioma (U251.dsRed) cells after 18 h of co-culture in Puramatrix. Figure 1 shows a Z-axis projection of a 102 µm-thick volume in which approximately 37% of total HB1.F3.eGFP NSCs were found in asymmetric contact with, or encircling, U251.dsRed glioma cells (Figure 1*A*, examples are shown circled in blue). We confirmed that these were true contacts (rather than projection superpositions) by also examining X-Z and Y-Z projections as shown for the cell pair indicated (Figure 1*B*). These HB1.F3.eGFP::U251.dsRed pairings were reminiscent of the close NSC-tumor cell contacts previously described *in vivo* [1], [10], [11], [24], [25].

The remaining 53% of HB1.F3.eGFP cells not in contact with U251.dsRed cells displayed two characteristic morphologies. Approximately 60% remained spherical or ellipsoid, possibly with some small soma extensions into the matrix, while ∼16% showed extended morphologies with one or two emerging processes often bifurcated at their leading edge. These appear to represent HB1.F3.eGFP cells migrating through the Puramatrix.

The U251.dsRed glioma cells were primarily spherical or elongated (approximately 83%), with some extending short processes (approximately 16%), but very few (approximately 2%) displaying long filopodia-tipped processes. They were never observed encircling HB1.F3.eGFP NSCs.

**Figure 2:**
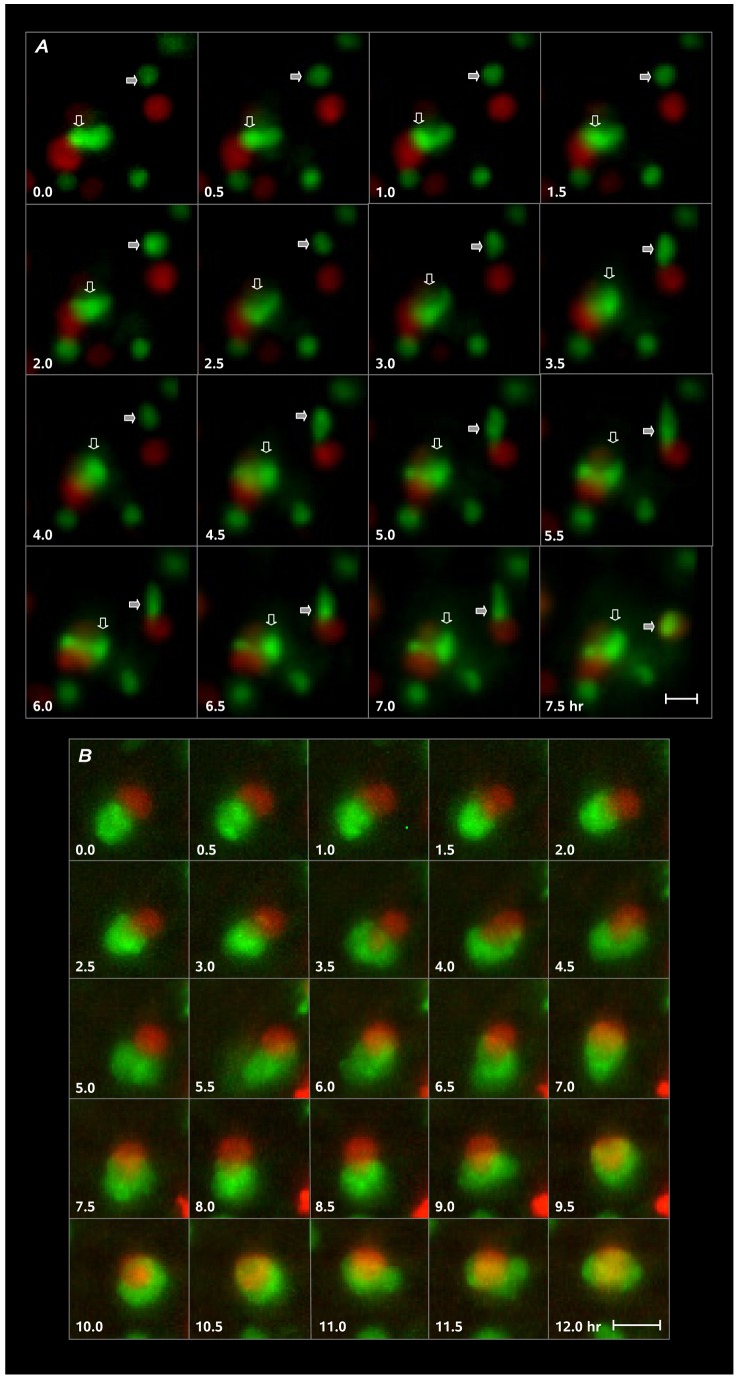
HB1.F3.eGFP cell movement and encirclement of U251.dsRed target cells. Frames are excised from larger wide-field sequences taken at 30 min intervals. ***A***, Approach and contact formation by one HB1.F3.eGFP cell (filled arrow) and progression of contact formation by another (open arrow). Stages of HB1.F3.eGFP cell (filled arrow) approach and contact formation were typical: extension of a leading process making contact with the U251.dsRed cell (3.5 h) followed by translocation of the cell body (5.0–7.0 h) and then envelopment of the glioma cell (7.5 h). The other HB1.F3.eGFP cell (open arrow), initially in contact with one of two adjacent U251.dsRed cells, over time extended forward and laterally to move off the surface of both cells. A third HB1.F3.eGFP cell (lower right, not marked) appeared to contact the U251.dsRed cell of interest to the second HB1.F3.eGFP cell, begin to move over the U251.dsRed cell surface (1.0–3.0 h), stall and regress (3.5–7.0 h), and then begin to advance at the end of the sequence (7.5 h). ***B***, Process of HB1.F3.eGFP cell encirclement of an U251.dsRed.dsRed target. Lamellipodia extend from the initial contact site (1.0–3.0 h) over almost the entire surface of the U251.dsRed glioma cell (6.0–12.0 h) to eventually encircle the U251.dsRed cell. Scale bars  = 15 µm.

Cell movements establishing HB1.F3.eGFP contact and encirclement of U251.dsRed cells were further examined by time-lapse imaging. In common with other observations of pre- and postnatal neural progenitor cells under more physiological conditions [28], [29], HB1.F3.eGFP NSC behaviors were heterogeneous, with subsets of these cells actively moving while others were more stationary, extending process and probing their immediate environment. The sequence in Figure 2*A* tracks two active HB1.F3.eGFP cells in images acquired at 30 min intervals. One NSC (solid arrow) moving toward and contacting a U251.dsRed cell, exhibited episodic movement: extension of a leading process (3.0 to 4.5 h) followed by forward displacement of the cell soma (5.0 to 7.0 h), eventually culminating in retraction of the trailing process and encirclement of the target U251.dsRed cell. The other NSC (hollow arrow) in contact with a U251.dsRed cell at the beginning of the sequence, extended a process across the target cell (4.0 to 7.5 h). The second image sequence in Figure 2B illustrates the time-course of encirclement. Here, initial contact is followed by extension and retraction of lamellipodia (1.5 to 9.0 h) and then progressive encirclement of the U251.dsRed glioma target (9.5 to 12.0 h).

**Figure 3:**
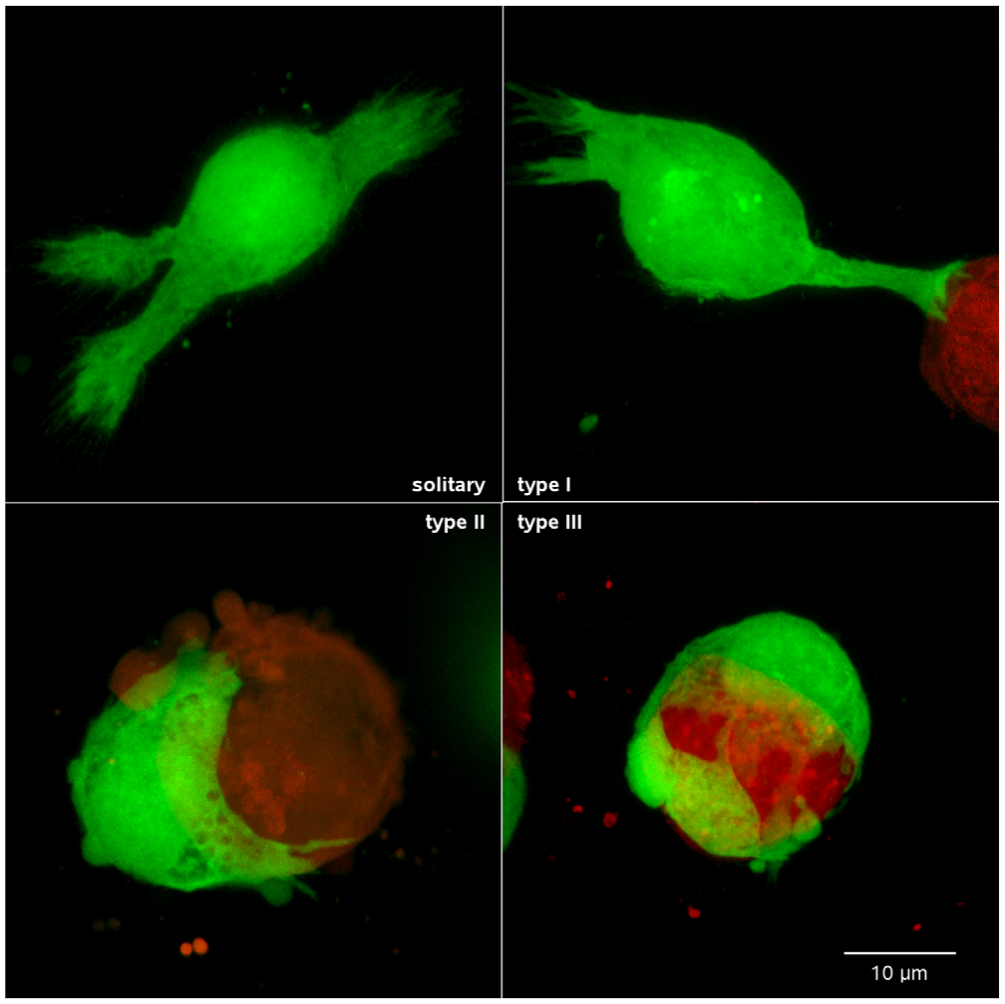
Stages of HB1.F3 contact formation and encirclement of U251.dsRed cells. Images are Z-axis projections of 28–44 optical sections taken at 1 µm intervals. ***A***, Solitary HB1.F3.eGFP cell extending filopodia-tipped process into the hydrogel matrix. ***B***, Initial HB1.F3.eGFP contact with a U251.dsRed cell (**Type I**). Note the morphology of the process contacting the target cell, which has shorter stubbier extensions resembling a holdfast. ***C***, Loss of processes, apposition of the HB1.F3.eGFP cell body to the U251.dsRed cell, and the beginnings of lamellipodia extension (**Type II**). ***D***, Complete HB1.F3.eGFP cell encirclement of a U251.dsRed cell with the cell body and lamellipodia covering much of the glioma cell surface (**Type III**).

This sequence of contact and encirclement was also examined in higher resolution images of fixed cells in Puramatrix, and divided into three stages based on morphology (Figure 3). In the solitary phase (Figure 3*A*), HB1.F3.eGFP cells extended filopodia-tipped process as if grabbing onto the surrounding matrix. In the type I contact, the HB1.F3.eGFP cell process is touching the U251.dsRed cell and extending finger-like projections over its surface (Figure 3*B*). In the type II contact, the HB1.F3.eGFP cell soma is now apposed to the target cell and lamellipodia are extending over its surface (Figure 3*C*), eventually progressing to encirclement of the U251.dsRed target (type III) (Figure 3*D*).

### Is there a preference for target cells by HB1.F3 neural stem cells?

We next examined whether the development of HB1.F3 NSC contacts varied between different types of target cells. To evaluate alternative brain tumor targets, HB1.F3.eGFP NSCs were presented with established human glioma lines (U251.dsRed, U87, D566) and a series of patient-derived brain tumor lines [30] expanded under stem cell culture conditions (PBT006, PBT022, PBT024) or subcutaneously for 4 or 14 passages prior to stem cell medium culture (PBT003 scp4, PBT017 scp4, PBT017 scp14). To compare contact formation between glioblastoma and another malignant tumor type, we evaluated breast cancer lines (MCF-7, SK-BR3, MDA-MB-231) as targets. To compare interactions between malignant and non-malignant target cells we utilized a human fibroblast cell line (3T3), primary mouse and human astroglial cells, and primary human dermal fibroblasts from adult and neonatal tissue.

**Figure 4:**
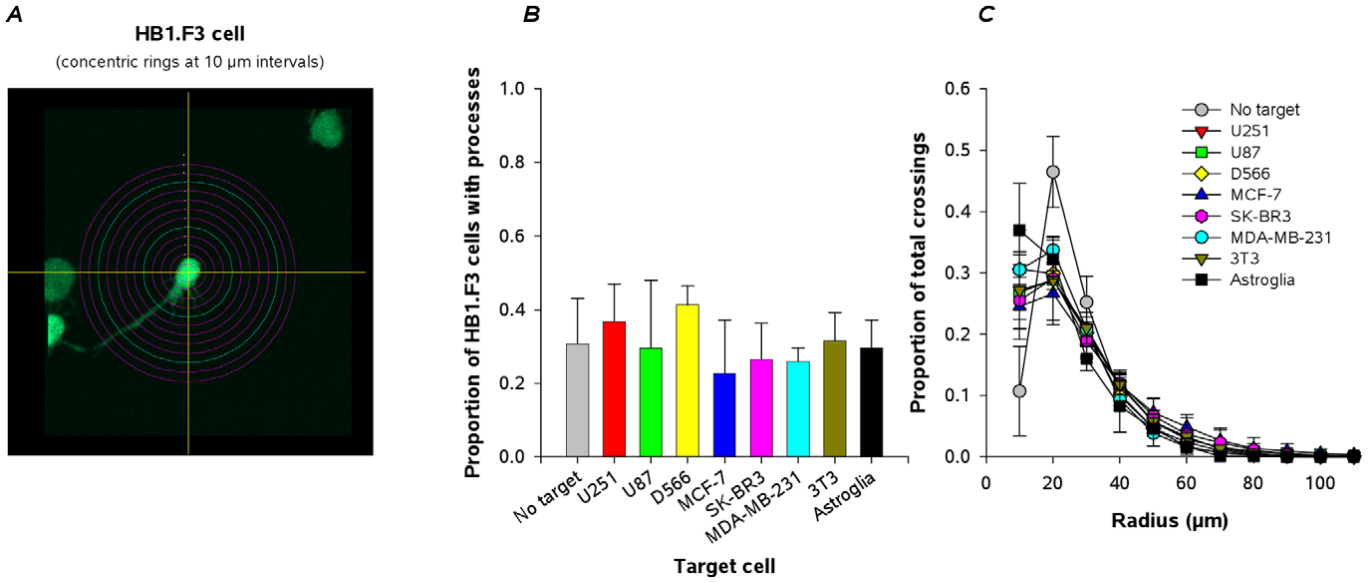
Process extension by solitary HB1.F3.eGFP cells does not vary with target cell type. ***A***, Illustration of Scholl analysis with concentric rings at 10 µm spacing centered and superimposed on the HB1.F3.eGFP cell soma. ***B***, Proportions of HB1.F3.eGFP cells exhibiting processes after culture in the absence of target cells, and after co-culture with potential target cells: glioma (U251, U87, D556); breast cancer (MCF-7, SK-BR3, MDA-MB-231); fibroblast (3T3), primary mouse astroglial cells. No significant differences (all *p*>0.05) were seen between target cells. ***C***, Lengths of HB1.F3.eGFP cell process after co-culture with each of the target cells indicated are virtually identical. Data are mean ± s.d. for 3 fields for 2–4 independent experiments.

Because solitary HB1.F3.eGFP cells extended filopodia-tipped process into the Puramatrix, we first asked if process extension varied with the potential cell target which could indicate conditioning influences of target cell-secreted factors (gradients being unlikely given the homogeneous mixtures of HB1.F3.eGFP and target cells). Using Sholl analysis, we evaluated solitary HB1.F3.eGFP cells for the numbers and lengths of extended process (Figure 4*A*) after 18 h in culture. For HB1.F3 cells grown in the absence of targets, or in the presence of six glioma and breast cancer lines, as well as with fibroblasts and mouse astroglia as targets, the proportion of total HB1.F3.eGFP cells bearing processes varied between 30% and 40%, with no significant differences observed (Figure 4*B*). Further, the lengths of these processes did not vary between target cell types (Figure 4*C*), with half the process being shorter than 40–50 µm.

We then asked if there were differences in the proportions of total HB1.F3.eGFP cells that formed contacts with different potential types of target cells. For these analyses each HB1.F3.eGFP and each target cell within multiple volumes of 900×900×102 µm were scored for potential interactions, our metric being the percentage of total HB1.F3.eGFP cells contacting a target cell (virtually all interactions were pair-wise). Within each volume scored, there were 103.5±37.9 HB1.F3.eGFP cells and 242.7±111.6 target cells (209 volumes in 68 cultures). We confirmed that variations in the relative proportions of HB1.F3.eGFP NSCs and target cells in each volume did not significantly affect formation of HB1.F3.eGFP::target contacts [ratios of target to HB1.F3.eGFP cells  = 2.9±1.9 for 45 volumes in 15 cultures (not shown)].

**Figure 5:**
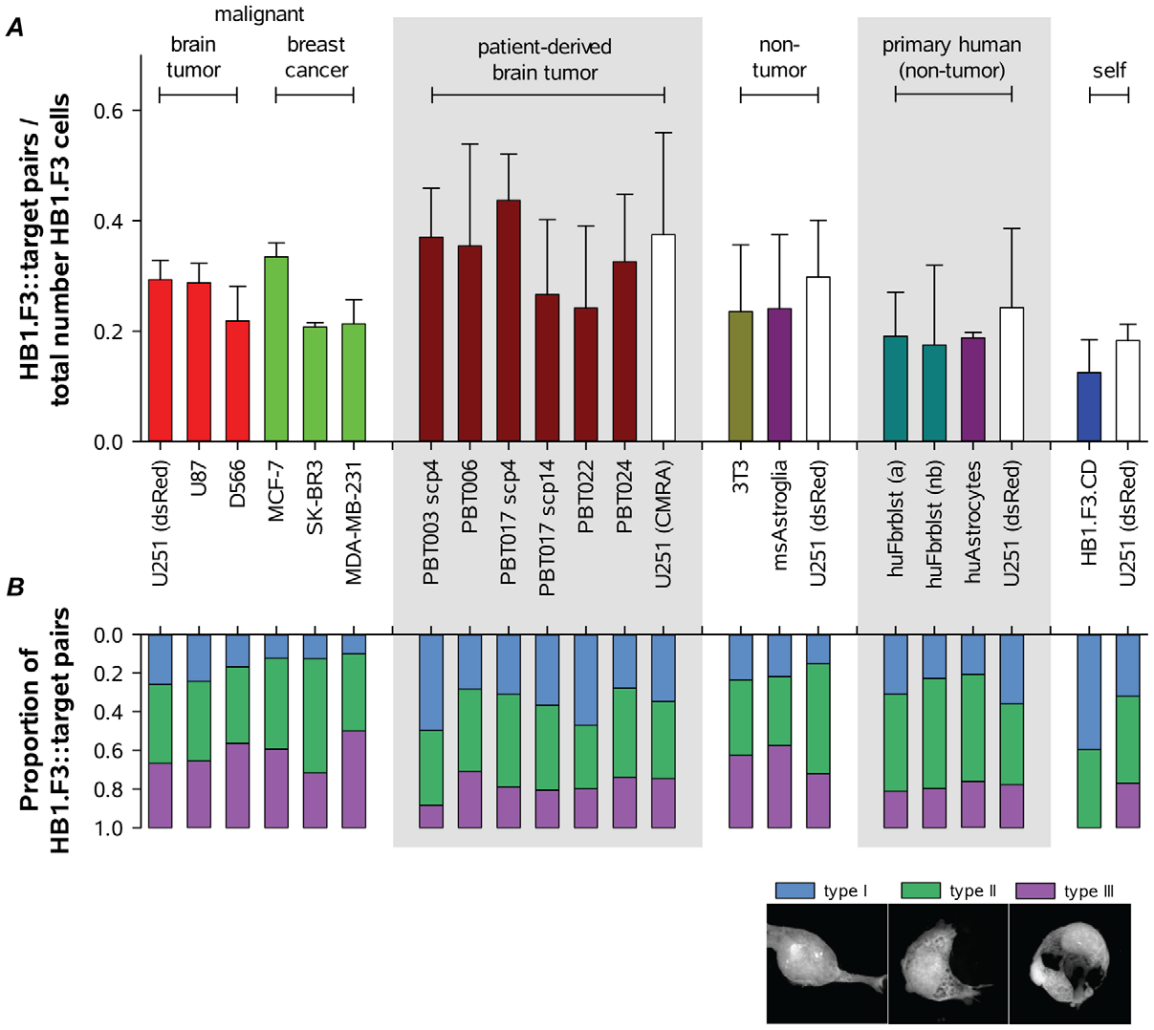
Analyses of HB1.F3.eGFP::target cell contacts. Formation of contacts, and the extent of target cell encirclement, did not vary with types of cell encountered, including multiple human malignant lines, primary human glioma cell lines, primary mouse astroglia, and human dermal fibroblasts (adult and new born) and astrocytes. ***A***, Proportions of total HB1.F3.eGFP cells contacting target cells. U251 cells were one of several malignant brain and breast cancer lines analyzed, and in addition served as a reference cell line for each additional group (marked by either dsRed or CMRA as indicated). No significant differences were observed (all *p*>0.05 within each group of target cells and across multiple groups; ANOVA with Bonferroni's multiple comparisons test, or *t* test, as appropriate). ***B***, Proportions of HB1.F3::target cell pairs displaying each of the thee morphologies defined as in Figure 3 and illustrated in the legend (showing only the HB1.F3.eGFP cell). No significant differences were observed (all *p*>0.05) with the exception of HB1.F3 homotypic interactions (see text). Data are mean ± s.d. for 3 or more volumes within each of 2–4 independent experiments.

As illustrated in Figure 5*A*, under assay conditions differing only in the type of target cell, 25–35% of HB1.F3.eGFP cells formed contacts. Note that these assays could not all be performed simultaneously, and therefore to provide a consistent reference population U251 cells were included for each examination of non-malignant target cells. Remarkably, there were no significant quantitative differences in contact formation between HB1.F3.eGFP cells and malignant established glioma and breast cancer lines, patient-derived brain tumor lines, a non-malignant fibroblast line, primary mouse or human astroglial cells, and primary adult or neonatal human dermal fibroblasts.

We then considered the possibility that while initial contact formation between HB1.F3.eGFP and target cells was a stochastic process initiated by surveying filopodia, progression to encirclement by HB1.F3.eGFP cells might vary between different types of target cells. Each HB1.F3.eGFP-target interaction was therefore additionally scored as type I, II or III (as illustrated in Figure 3). Again, no statistically significant differences were observed when HB1.F3.eGFP-target cell contacts were separated based on morphological criteria (Figure 5*B*), suggesting that all heterotypic cell surfaces were equally permissive for HB1.F3.eGFP NSC encirclement.

**Figure 6:**
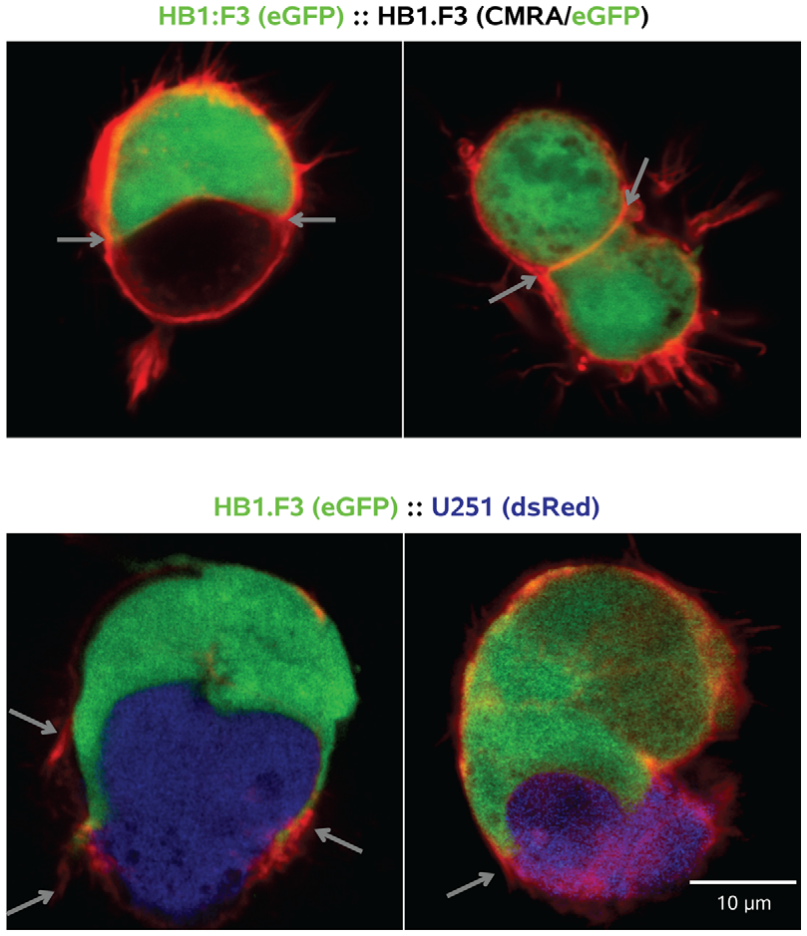
HB1.F3.eGFP cells will form contacts with, but not encircle, other HB1.F3 cells. *Above*, Images of HB1.F3.eGFP::HB1.F3.eGFP and HB1.F3.eGFP::HB1.F3.CD (CMRA) contacting pairs. Cell boundaries are outlined using phalloidin_Alexa647_ to show the cortical F-actin cytoskeleton; contact zones are marked by arrows. While the HB1.F3 cells showed filopodial extensions over their hydrogel-exposed surfaces, the contact zone was smooth and clearly delineated by the phalloidin_Alexa647_. *Below*, Heterotypic HB1.F3.eGFP::U251.dsRed pairs were characterized by extension of actin-rich (arrows) lamellipodia from the HB1.F3.eGFP cell over the target glioma cell. These images illustrate the data presented quantitatively in Figure 5.

The only example of target cell discrimination we observed was for HB1.F3.eGFP cells contacting other HB1.F3 cells. These experiments were performed in the same manner as those probing heterotypic contacts, scoring HB1.F3.eGFP cell interactions with themselves or a separate target population of non-labeled HB1.F3 cells (HB1.F3.CD). As shown quantitatively in Figure 5*A*, while the proportions of HB1.F3.eGFP cells making contact with target cells were similar for HB1.F3.CD or contemporaneously-analyzed U251.dsRed targets, no HB1.F3.eGFP::HB1.F3.CD or HB1.F3.eGFP::HB1.F3.eGFP contacts progressed beyond soma-soma contact (type II) to encirclement (type III). This is illustrated in Figure 6, where F-actin bound by fluorescent phalloidin_Alexa647_ illuminates cell peripheries. Lamellipodia can be seen extending over the surface of the target U251.dsRed cell (arrows), while for HB1.F3.eGFP cells in contact with HB1.F3.CD or HB1.F3.eGFP targets a smooth phalloidin_Alexa647_-labeled boundary demarcates the area of cell-cell contact (arrows).

### Cytoskeletal involvement in HB1.F3 NSC movement and encirclement of U251 glioma cells

Extension of the leading process by migrating neural progenitor cells and immature neurons is often ascribed to microtubules [31], [32], although F-actin filaments have also been implicated [33]. We observed that disruption of microtubules with nocodazole (1–10 µM) prevented all but the most minimal process extension by HB1.F3.eGFP cells, and permitted formation of only rudimentary superficial contacts with U251.dsRed targets (not shown). However, nocodazole was also disproportionately toxic to HB1.F3.eGFP cells, making interpretation of these experiments problematic.

**Figure 7:**
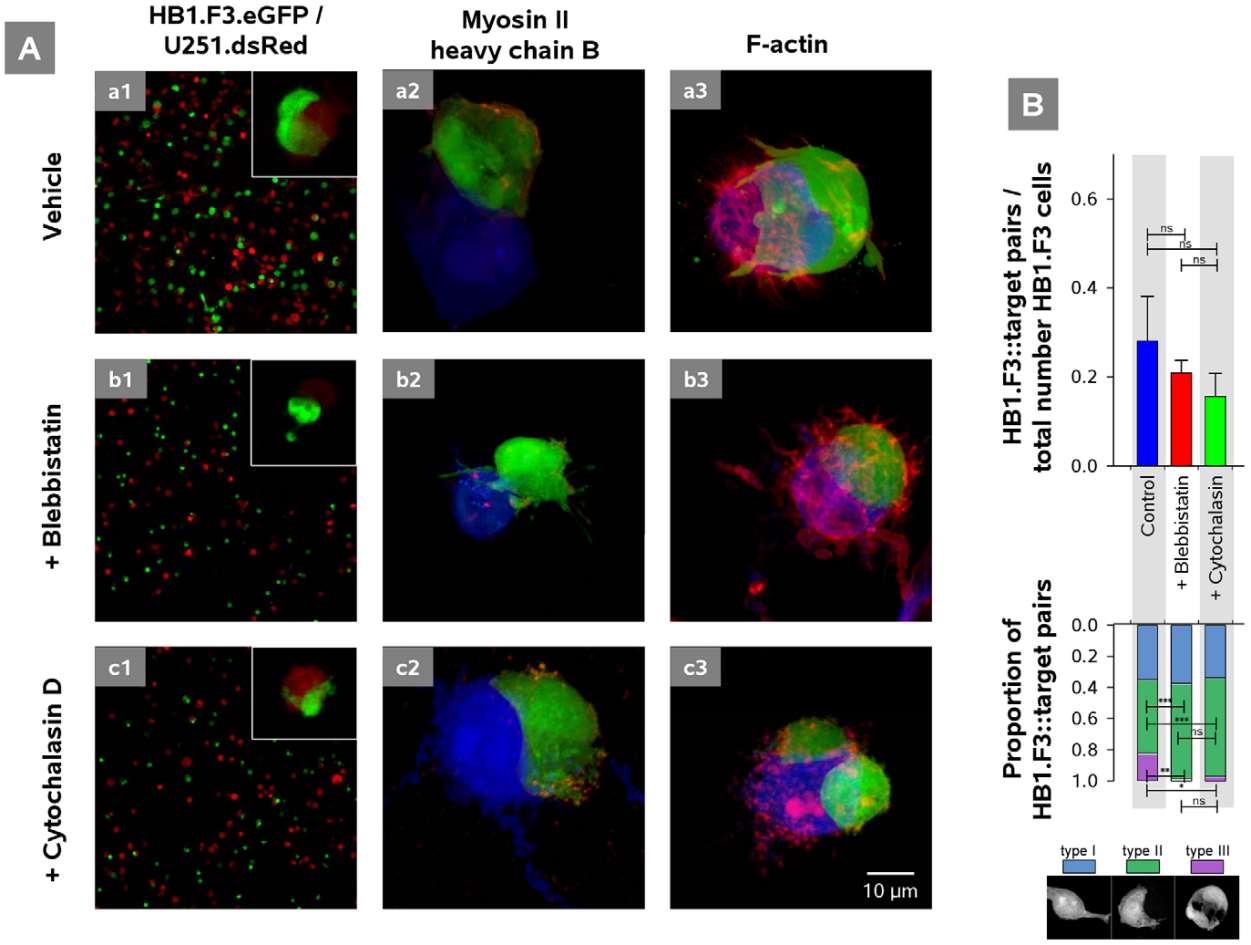
Disruption of F-actin filaments with cytochalasin D and myosin II by blebbistatin and consequences for motor protein distributions and HB1.F3.eGFP interaction with target U251.dsRed cells. ***A***, Images illustrating the sensitivities of cell morphology in Puramatrix (*a1, b1, c1*) and of the distributions of myosin II heavy chain B (by antibody immunofluorescence) (*a2, b2, c2*) and F-actin (by phalliodin_Alexa647_ fluorescence) (*a3, b3, c3*). ***a1***, Normal pattern of HB1.F3.eGFP::U251.dsRed cell interactions (inset shows U251.dsRed cell encirclement by a HB1.F3.eGFP cell). ***a2, a3***, Normal distributions of MHC-IIB and F-actin. ***b1***, Disruption of target cell encirclement after MHC-IIB inhibition by blebbistatin (50 µM; 18 h), contraction of MHC-IIB (***b2***) and formation of long actin-lined tubes emanating from both tumor cells and NSCs (***b3***). ***c1***, Loss of lamellipodia and truncation of target cell encirclement (inset) after F-actin inhibition by cytochalasin D (1 µM; 18 h), and loss of filamentous structure and appearance of multiple aggregates by both MHC-IIB (***c2***) and F-actin (***c3***) and loss of spike-like filopodial structures emerging from both HB1.F3.eGFP and U251.dsRed cells (***c3***). ***B***, *Above*, Proportions of HB1.F3.eGFP:: target cell pairs were not significantly affected by actin or myosin inhibition (*p*>0.05). *Below*, After actin disruption or myosin inhibition, encirclement was blocked and did not progress beyond type II (*p*<0.05).

Leading-edge filopodia and lamellipodia of migrating cells are highly dynamic actin-based structures in which force is generated by non-muscle myosin (myosin II) motor proteins [34], [35]. Distributions of myosin II isoforms IIA and IIB were examined by immunofluorescence using isoform-specific antibodies against myosin heavy chain (MHC), and F-actin distribution was imaged using phalloidin_Alexa647_. MHC-IIB immunoreactivity was stronger in HB1.F3 NSCs, and therefore we focused on this isoform rather than the MHC-IIA isoform expressed more strongly in U251 glioma cells (not shown, [36]). Figure 7*A* shows the normal distributions of these proteins in HB1.F3.eGFP NSCs during contact with U251.dsRed target cells, and their redistributions following pharmacological disruption. MHC-IIB under control conditions was found in a cortical band adjacent to the cell surface (Fig. 7*A* *a2*). F-actin was found in filopodia of both HB1.F3.eGFP and U251.dsRed cells, immediately under the membrane of extending processes in HB1.F3.eGFP cells, and in some cases as a peripheral rind just under somatic cell membrane (Figure 7*A* *a3*). Myosin II inhibition with blebbistatin (50 µM) resulted in disruption and contraction of subcortical MHC-IIB domains (Figure 7*A* *b2*) and the appearance of long tubular F-actin rich processes [37] on both HB1.F3.eGFP and U251.dsRed cells (Figure 7*A* *b3*). Disruption of F-actin with cytochalasin D (1 µM) was followed by appearance of MHC-IIB puncta at the edges of HB1.F3.eGFP cells (Figure 7*A* *c2*), and not-well-defined areas of F-actin aggregation in both HB1.F3.eGFP and U251.dsRed cells (Figure 7*A* *c3*).

The functional consequences of actin-myosin disruption were striking. Quantitatively, numbers of HB1.F3.eGFP cells contacting U251.dsRed targets were not significantly affected by blebbistatin or cytochalasin D, and this was reflected in the stability of the proportion of type I contacts between NSCs and targets (Figure 7*B*). However the progression from type II to type III contact was significantly impaired by both blebbistatin and cytochalasin D, consistent with the roles of myosin and actin in lamellipodial expansion (Figures 7*A* *b1* and 7*Ac1*).

## Discussion

Formation of contacts between HB1.F3 NSCs and glioma cells *in vivo* is a complex process involving both the tumor cells and their interactions with surrounding brain and tumor stroma to form an overall microenvironment. The experiments reported here were designed to investigate formation of contacts between HB1.F3 NSCs and target cells in a peptide hydrogel matrix in which contributions from the microenvironment are minimized, thereby allowing analysis of cell behaviors in relative isolation. We evaluated HB1.F3.eGFP contact and encirclement of target glioma and non-glioma tumor cell lines, a non-malignant cell line, patient-derived glioma cells, primary mouse and human astroglial cells, and primary human dermal fibroblasts. Our major conclusion is that outside of the brain, in the absence of any additional external cues, HB1.F3 NSC contact and subsequent encirclement of glioma cells and other non-self target cells appears to be an expression of intrinsic behaviors that are not directed at tumor cells per se. As illustrated in Figure 5, quantitative comparisons of HB1.F3 NSC contacts with target cells did not reveal significant preferences (with the notable exception of self-encirclement), indicating that many cell surfaces are permissive for contact formation. These observations therefore suggest that preferential formation of cell contacts *in vivo* (as well as long range migration which is not addressed in this study) involves contributions originating in the microenvironment created by tumor cells and surrounding brain.

It is important to note that while migratory behaviors of HB1.F3 cells appear to be cell-intrinsic, they are also quite plastic. Comparing 2- and 3-dimensional culture conditions, the differences between movements with a pattern of extension followed by soma translocation extension of HB1.F3 cells in the 3-dimensional Puramatrix environment described here and the predominately amoeboid movements exhibited by these same cells in 2-dimensional culture [16] (see [28], [36] for other examples) point to considerable sensitivity of these NSCs to variations in surrounding environmental cues. This flexibility in choice of migratory mechanisms could be reflected in the two distinct phases of HB1.F3 NSC movements observed here: locomotion in Puramatrix and encirclement of target cells. We noted that the intermittent movements of HB1.F3 cells in Puramatrix were reminiscent of neural progenitor and immature neuron migration during cortical development [29], [38], as well as to glial precursor movements and glioma cell dissemination [36], [39]–[41]. While differing in mechanistic detail, cell movements in all these systems show a thematically common pattern of leading process extension followed by soma translocation. Subsequent formation of target cell contacts by HB1.F3 cells and initiation of encirclement appear to evoke a mechanistic program in which F-actin and myosin II interact to drive lamellipodia expansion, as in other instances of cell migration [42]. This transition from one migratory program to another in response to a change in the substrate [43] may be reflected in differential sensitivity to cell-surface signals that for HB1.F3::HB1.F3 interactions allow for formation of homotypic contacts that are not permissive for subsequent extension of lamellipodia.

Some tumor-tropic stem cells imaged *in vivo* in fixed tissue, including HB1.F3 NSCs, immortalized rat neural progenitor cells, mouse bone marrow-derived NSCs, mouse primary NSCs, and endogenous mouse neural precursor cells, appear adhered to tumor cells in preference to other brain cells [1], [8], [10], [11], [24], [25]. One possible model would have NSCs relatively immobilized once in tumor cell contact. However, these static images are only snapshots of dynamic processes, and they give no indication of the temporal stability of NSC-tumor cell contacts. In an alternative view, NSCs could be in constant motion, moving over a variety of permissive substrates during migration towards tumor sites. In this scenario, signals originating in the tumor microenvironment, in conjunction with tumor cell-derived signals, could stabilize an NSC swarm in and around tumor foci. Either way, the observations presented here point towards extensive engagement of NSCs with the tumor microenvironment, the element missing in these hydrogel-based cultures in promoting selective contacts between NSCs and tumor cells. Cell surface components within tumor cell niches, including extracellular matrix (ECM) associated with vasculature and axon tracts, soluble signals originating in the brain parenchyma stimulated by the presence of tumor cells or tumor stroma, along with the plethora of other reciprocal tumor-brain interactions established at tumor foci, appear in the aggregate to create a microenvironment favoring HB1.F3 proximity to glioma cells.

It has been suggested that essentially all glioma cells will have to be eliminated will be required to achieve curative efficacy in patients [44], and given the disseminated nature of high-grade glioma, cell-based therapies incorporating autonomous tracking of tumor cells are a promising strategy. Therefore, understanding the details of therapeutic and target cell interactions will be important for optimizing therapeutic designs. The investigation presented here separates processes of contact between NSCs and tumor cells from their brain milieu, in an effort toward this goal for NSC-based cancer therapies [4], [6]. The properties of HB1.F3 NSCs and their therapeutic potential have been studied to a greater extent than other NSCs. In summary tables of experimental investigations of potential NSC-based therapies, 5 of 13 studies compiled by Ahmed et al. [45] and 10 of 18 listed by Kim [6] involve HB1, F3 cells, and, as noted, they are currently in clinical trial [7]. Because of their advanced development as therapeutic cells, understanding details of cell-cell interactions by HB1.F3 NSCs has the potential to directly further clinical development by, for example, manipulation of their intrinsic properties. An example of how this might occur is provided by Jurvansuu et al. [46], who demonstrated that up-regulation of cytokine receptor expression in NSC lines enhanced migration efficiency.

More generally, it has been widely noted that migration towards brain tumors is a property shared by many types of stem cells, including adult, neural progenitor and embryonic stem cell-derived NSCs [1] (for reviews see [4], [6]) and mesenchymal stem cells (MSCs) of multiple origins [47] (for reviews see [48], [49]). While there have been few direct comparisons of potentially therapeutic NSCs and MSCs [50]–[52], differences in migration and other behaviors may exist as consequences of underlying signaling pathways that will vary between stem cells of different lineages. For example, subtle distinctions have been noted between embryonic stem cell-derived and somatic NSCs in differentiation potential and proliferation [53], and MSCs of different origins differ in proliferation, differentiation potential and tumor-homing capabilities [54]–[56]. In addition, a difference in the capacity of NSC and MSC lines to deliver a therapeutic oncolytic adenovirus payload has been reported [52]. Ultimately, these variations in the specific properties of particular therapeutic NSCs, MSCs or other cells will impact treatment efficacy and therefore influence the design of these stem cell-based therapies.

## Materials and Methods

### Culture of HB1.F3.eGFP cells and target cells

The human NSC line HB1.F3 was derived from 15-week human fetal telencephalon and immortalized by v-*myc* expression [57], [58]. HB1.F3.CD and HB1.F3 NSCs expressing eGFP (HB1.F3.eGFP) cells were cultured in DMEM (high glucose; Gibco 11960-044) supplemented with 10% heat inactivated fetal bovine serum (Omega Scientific FB-02), 1% L-glutamine (Gibco 25030-081) and 1% penicillin-streptomycin (Hyclone SV30010). Target glioma (U251, Abgent; U251.dsRed, our laboratory; U87, ATCC; D566, Darell Bigner) [59], [60], breast (MCF-7, ATCC; SKBR3, ATCC; MDA.MB.231, ATCC) [61] and fibroblast (NIH 3T3, ATCC) [62] cell lines, and primary human dermal fibroblast (adult and neonatal, Invitrogen C-013-5C; C-004-5C) cells, were cultured in this same medium. Target patient-derived brain tumor lines PBT003, PBT006, PBT017, PBT022 and PBT024 [30] were expanded as tumor spheres in DMEM/F-12 50∶50 v/v (MediaTech, Inc. 15-090-CV) plus 1% L-glutamine (Gibco 25030-081), 1% penicillin-streptomycin (Hyclone SV30010), 2% B-27 (Gibco 17504-044), 25 mM HEPES (Irvine Scientific 9313), 1,000 U/ml heparin sodium (American Pharmaceutical Partners 401811B), and supplemented 2x weekly with 20 ng/ml EGF (R&D Systems 236-EG) and 20 ng/ml FGF (R&D Systems 233-FB). Tumor spheres were passaged using 20 µg/ml Accutase (Innovative Technologies AT104) when the media's pH reached 6.8. Primary mouse astroglial cell cultures were prepared from P1-P4 pan-mRFP mice [13]. The telencephalon was dissected, enzymatically dissociated (papain, per manufacturer's instructions; Worthington Biochemical 3160) and cultured in MEM (Gibco 51200) plus 10% FBS, 1% L-glutamine and 1% penicillin-streptomycin. Primary human astrocytes (Cell Applications 882K) were cultured in Astrocyte Growth Medium (Cell Applications 821–500) supplemented with 10% Astrocyte Growth Supplement (821-GS).

Target cells not genetically-marked were labeled using CellTracker Orange CMRA (Invitrogen C34551). Enzymatically dissociated cells were pelleted by centrifugation, resuspended in pre-warmed CellTracker-containing (5 µM) serum-free medium (Gibco 11960-044), and loaded for 40 min at 37°C in the culture incubator. Cells were then pelleted, resuspended in growth medium (DMEM, 10% FBS, 1% L-glutamine, 1% penicillin-streptomycin), incubated for 30 minutes in a 37°C water bath, rinsed (2x) in 10% sucrose in tissue culture-grade water (as for all solutions contacting living cells), and added to Puramatrix at the desired concentration. The marking method did not appear to influence formation of HB1.F3 contacts; results obtained using U251 cells marked by dsRed expression or by CMRA were indistinguishable in the assays used here (see results in Figure 5*A*).

### NSC-target cell co-cultures

Puramatrix, a self-assembling synthetic peptide hydrogel [27] (3DM, Cambridge, MA) (BD 354250) was prepared by sonicating 1% Puramatrix stock solution (30 min), and diluting with 10% sucrose to a final working concentration of 0.3%. For each experiment, HB1.F3.eGFP cells and target cells were trypsinized (Gibco 25200-056) and resuspended separately in growth medium at 2×10^6^ cells/ml, mixed together 1∶1 v/v, rinsed 1x in PBS (Gibco 14190-144), pelleted, and resuspended in 10% sucrose solution at 4×10^6^ cells/ml. Equal volumes of the HB1.F3.eGFP/target cell mixture and 0.3% Puramatrix working solution were then mixed together, and 150 µl was pipetted using a clipped 200 µl tip into 1 ml growth medium per well of a 24-well plate to a final cell density of 1.5×10^5^ total cells/ml. For cytoskeletal disruption, the HB1.F3.eGFP and target cell mixture was encapsulated in growth medium containing either blebbistatin (Sigma B0560) or cytochalasin D (Sigma C8376). In all cases cells were grown for 18 h in a 5% CO2 atmosphere at 37°C. For imaging and then scoring of HB1.F3.eGFP-target cell interactions, growth medium was removed, and the cell-containing Puramatrix gel was fixed (4% paraformaldehyde in Tris-buffered saline (TBS) overnight at 4°C, and mounted in Prolong Gold (Invitrogen P36930) in a depression slide.

### Immunostaining

Primary antibodies and labeled toxins were obtained from commercial sources: Alexa647-conjugated phalloidin (phalloidin_Alexa647_) (1∶1000, Invitrogen A22287); anti-myosin heavy chain (MHC)-A (1∶500, Covance PRB-440P); anti-MHC-B (1∶500, Covance PRB-445P).After fixation, Puramatrix cultures were rinsed (1x) with wash solution (TBS +0.1% Triton X-100), blocked using a 1∶1 v/v mixture of Western Blocking Reagent (Roche 11921681001) and BlockAid solution (Invitrogen B10710) plus 1% Triton X-100 for 1 h at room temperature, incubated in toxin or primary Ab diluted into blocking solution overnight at 4°C, rinsed 3x with wash solution, exposed to secondary Ab with host and target species as appropriate (all Alexa fluorophores from Invitrogen, 1∶1000 in blocking solution) for 3 h at room temperature, rinsed 3x with wash solution, and mounted in Prolong Gold on depression slides.

### Imaging and quantitative analyses

For each Puramatrix gel, three volumes of 900×900×102 µm (34 Z-axis steps at 3 µm separation) were sampled by confocal microscopy (Zeiss 510) using a 10x Fluar (0.5 NA) objective. Contacts were scored from image stacks and confirmed by examination of adjacent optical sections. For the analysis of HB1.F3 homotypic interactions, populations of eGFP-expressing NSCs and non-eGFP-engineered cytosine deaminase-expressing HB1.F3 (HB1.F3.CD) cells were co-cultured, fixed, and treated with phalloidin_Alexa647_ to label cortical actin and outline HB1.F3 populations for scoring of cell-cell interactions.

### Time lapse imaging

Cultures of HB1.F3.eGFP and U251.dsRed cells in Puramatrix were grown in an environmental chamber on a Zeiss AxioObserver inverted microscope. Images were collected at 30 min intervals for up to 18 h using a Hamamatsu EMCCD camera. Image sequences were filtered, adjusted for brightness and contrast, and cropped around regions of interacting cells using ImagePro 6.3 (MediaCybernetics).

### Statistical analyses

All data are presented as mean ± s.d. as indicated in the figure legends. Tests for statistical significance in Figures 4, 5 and 7 were one-way ANOVA followed by Tukey's multiple comparisons test (Prism 5; GraphPad Software). Data were considered significant for *p*<0.05.
